# Supratentorial meningeal melanocytoma mimicking meningioma: case report and literature review

**DOI:** 10.3389/pore.2023.1611482

**Published:** 2024-01-04

**Authors:** Mayle Gomes Ferreira de Araújo, Luiz Euripedes Almondes Santana Lemos, Pedro Lucas Negromonte Guerra, Fernanda Marcia dos Santos Lima Didjurgeit, Auricelio Batista Cezar, Igor Vilela Faquini, Hildo Rocha Cirne de Azevedo Filho

**Affiliations:** ^1^ Department of Neurosurgery, Hospital da Restauração, Recife, Brazil; ^2^ Center for Medical Sciences, Universidade Federal de Pernambuco, Recife, Brazil

**Keywords:** primary melanocytic tumor, melanocytoma, brain tumor, primary brain tumor, primary meningeal melanocytomas

## Abstract

**Introduction:** Primary melanocytic tumors originating from CNS melanocytes are rare, with a low incidence of 0.7 cases per 10 million annually. This study focuses on primary leptomeningeal melanocytomas, emphasizing their epidemiology, clinical characteristics, and diagnostic challenges. Despite their infrequency, these tumors warrant attention due to their unique features and potential for local recurrence.

**Case Report:** A 32-year-old female presented with syncope and seizures, leading to the discovery of two left-sided supratentorial lesions initially misidentified as convexity meningiomas. Detailed imaging suggested meningioma-like features, but intraoperative findings revealed unexpected hyperpigmented lesions. Histopathological examination, supported by immunohistochemistry, confirmed primary leptomeningeal melanocytoma. The surgical approach and subsequent management are discussed.

**Discussion:** The discussion emphasizes challenges in diagnosing primary leptomeningeal melanocytomas. Treatment debates, especially regarding adjuvant radiotherapy, are explored. Recurrence risks stress the importance of vigilant follow-up, advocating for complete surgical resection as the primary approach. The rarity of supratentorial cases adds complexity to diagnosis, necessitating a multidisciplinary approach. Insights from this case contribute to understanding and managing primary leptomeningeal melanocytomas, addressing challenges in differentiation from more common tumors and prompting ongoing research for refined diagnostics and optimized treatments.

**Conclusion:** This study contributes insights into primary leptomeningeal melanocytomas, highlighting their rarity in supratentorial regions. The case underscores the importance of a multidisciplinary approach, incorporating clinical, radiological, and histopathological expertise for accurate diagnosis and tailored management. Ongoing research is crucial to refine treatment strategies, enhance prognostic precision, and improve outcomes for individuals with this uncommon CNS neoplasm.

## Introduction

Primary melanocytic tumors of the central nervous system (CNS) are lesions derived from melanocytes and were first described in 1859 by Rudolf Virchow [1]. In the already-formed CNS, these cells are found in the leptomeninges (arachnoid and pia mater), with their highest concentration in the upper portion of the spinal cord [2].

Unlike secondary melanomas that affect the CNS, which have a high incidence, primary melanocytic tumors of the CNS are rare, with an incidence of 0.7 cases per 10 million inhabitants per year [3–5].

In the latest classification of CNS neoplasms by the World Health Organization (WHO) in 2021, primary CNS melanocytic tumors were categorized into four types (Table 1): primary leptomeningeal melanoma, primary leptomeningeal melanocytoma, primary leptomeningeal melanomatosis, and primary leptomeningeal melanocytosis [9].

**TABLE 1:** Primary melanocytic tumors of the CNS - WHO, 2021.

Type	Incidence	Macroscope appearance	Histological characteristics	Location
Primary leptomeningeal melanoma	0,7 cases/10,000,000 people/year [6]	Solitary solid black lesion and extra-axial	High cell density, high mitosis rate varied cellular arrangement, great number of atypical mitotic figures and invasion of adjacent tissue	Posterior fossa and medulla
Primary leptomeningeal melanocytoma	1 cases/10,000,000 people/year [5]	Solitary, extra-axial mass of varied colour	Low mitosis rate, little atypia well-differentiated cells without parenchymal invasion	Medulla
Primary leptomeningeal melanocytoma	1 cases/10,000,000 people/year [7]	Solid, black and diffuse	High mitosis rate, high cell density, nuclear atypia and parenchymal invasion	Posterior fossa and medulla
Primary leptomeningeal melanocytoma	1 cases/10,000,000 people/year [8]	Black and diffuse lesion without macroscopic formation	Low mitosis rate, variably shaped cells, no atypia and no signs of parenchymal invasion	Temporal lobe, brainstem and posterior fossa

Among these, primary leptomeningeal melanocytomas (the focus of this study) are benign neoplasms with a low incidence in the population, affecting less than one patient in every 10 million per year. Unlike other primary melanocytic tumors of the leptomeninges, they are more common in women, ranging in age from 20 to 80 years, with a peak incidence in the population aged between 40 and 50 years [5].

It is a benign tumor that can be found anywhere in the CNS, most commonly affecting the spinal cord, especially the cervical spine, and the posterior fossa, with a supratentorial location being less common [10]. The intracranial areas most commonly involved are the skull base, the cerebellopontine angle, the pineal region, and the Cavum of Meckel. Two cases of lesions in the convexity region that mimicked convexity meningiomas have been described, one in 2012 by Lin et al. [11] and the other in 2006 by Beseoglu et al. [10].

These are solitary lesions, usually compressing and not invading adjacent brain tissue. Clinical manifestations are caused by increased intracranial pressure, leading the patient to suffer from seizures, migraines, subarachnoid hemorrhages, and focal neurological deficits [12].

The prognosis for these patients is generally favorable, but local recurrence is not uncommon [10, 13, 14]. Complete surgical resection of the lesion is the recommended treatment. Some authors suggest adjuvant radiotherapy in cases of both complete and partial resection of the lesion [13, 15]. Immunohistochemical studies show the expression of melanocytic markers, such as the S-100 protein, Melan-A, and HMB-45, and the absence of meningothelial markers, such as EMA [13].

In this paper, we report a case of a 32 year-old female patient with a melanocytoma confirmed by histology and immunohistochemistry, who had two lesions that mimicked a left convexity meningioma. We also review the literature on this uncommon pathology, presenting an extensive differential diagnosis.

## Case report

### Clinical case

A 32 year-old female patient was admitted to the neurosurgery department of Hospital da Restauração with a history of syncope and seizures 2 months prior to admission. During the physical examination, she exhibited a Glasgow Coma Scale (GCS) score of 15 and had no motor deficits. Notably, the patient had a hyper-pigmented nevus on the left eye, which she referred to as a birthmark.

**FIGURE 1:**
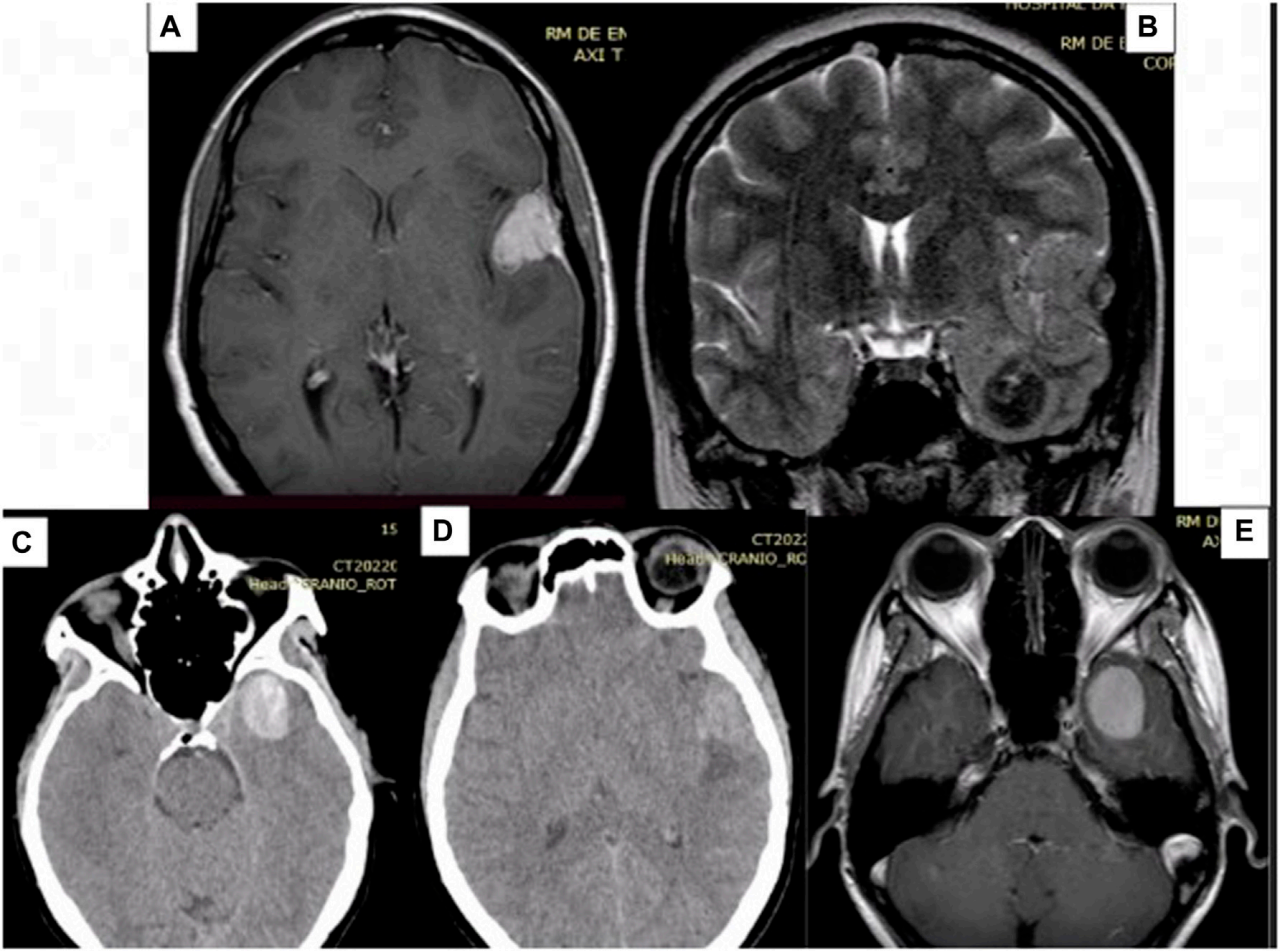
Preoperative MRI images showing two lesions, one on the floor of the middle fossa and the other in the pterygium region, both on the left. **(A)** Contrast-enhanced T1-weighted MRI of the pterional lesion, which appears hyper-intense and with homogeneous contrast uptake. **(B)** Coronal T2 MRI showing the two lesions. **(C)** Axial CT scan, without contrast, showing that the deeper lesion is hyper-dense in relation to the parenchyma. **(D)** Axial CT scan without contrast. **(E)** Contrast-enhanced T1-weighted MRI of the deepest lesion, which appears hyper-intense and with homogeneous contrast uptake. MRI: Magnetic Resonance Imaging. CT: Computed Tomography.

A CT scan of the skull revealed two lesions (see Figure 1): one on the floor of the left middle fossa and the other on the lateral third of the lesser wing of the sphenoid (pterional), both on the left side. Initially, the possibility of intracranial hematoma was considered. Given the patient’s age and the locations of the lesions, a vascular study was initiated, involving cerebral arteriography and a brain MRI. The arteriography revealed no vascular alterations.

The brain MRI (see Figure 1) indicated that the presumed pterional lesion was extra-axial and had dural implantation in the left temporal convexity, suggesting the presence of a convexity meningioma. In the T1 sequence, both lesions exhibited hypersignal, along with homogeneous contrast enhancement and evidence of a broad dural implantation base (dural tail). On the T2 sequence, the temporal lesion showed hyposignal, while the other lesion displayed isointense characteristics, with discrete surrounding edema.

### Surgery

The surgical approach employed was an extended pterional approach to the floor of the left middle fossa. Following the craniotomy, a blackened appearance of the dura mater over the temporal lobe was observed.

**FIGURE 2:**
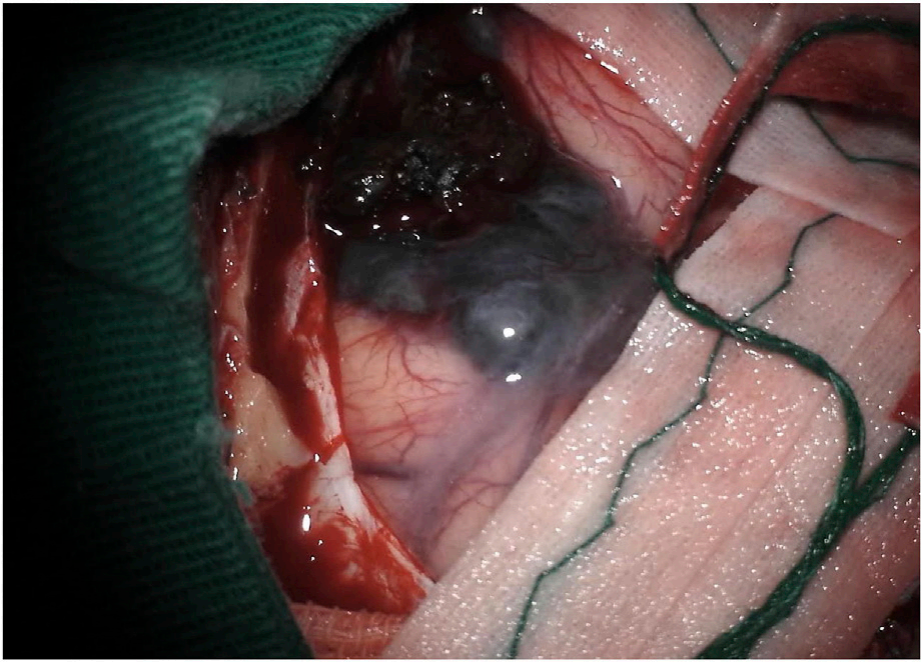
Intraoperative image showing a hyper-pigmented lesion.

Upon opening the dura, it became apparent that the presumed pterional lesion was not a meningioma but rather a hyperpigmented, softened, extra-axial, hemorrhagic lesion. This lesion exhibited pigment diffusion through the adjacent arachnoid (see Figure 2) and displayed a favorable cleavage plane with the adjacent parenchyma.

The resection began with the most superficial lesion, followed by dissection through the surgical cavity to access the second lesion. Subsequently, it was discovered that the second lesion was a blood clot.

Following the surgery, the patient was extubated and transferred to the intensive care unit. She experienced favorable progress during the post-operative period, maintaining a Glasgow Coma Scale (GCS) score of 15 without any deficits. She was discharged 4 days after the surgery. An immediate post-operative CT scan showed complete resection of both lesions.

After the procedure, considering the possibility of melanoma metastasis due to its higher incidence, a comprehensive investigation was conducted, including contrasted chest and abdominal examinations. These investigations did not reveal any primary or secondary neoplastic lesions. A dermatologist conducted a thorough body inspection and identified only a small periumbilical skin lesion on the left. This lesion was excised, and its histological report confirmed it to be an intradermal melanocytic nevus.

### Histopathological findings

**FIGURE 3:**
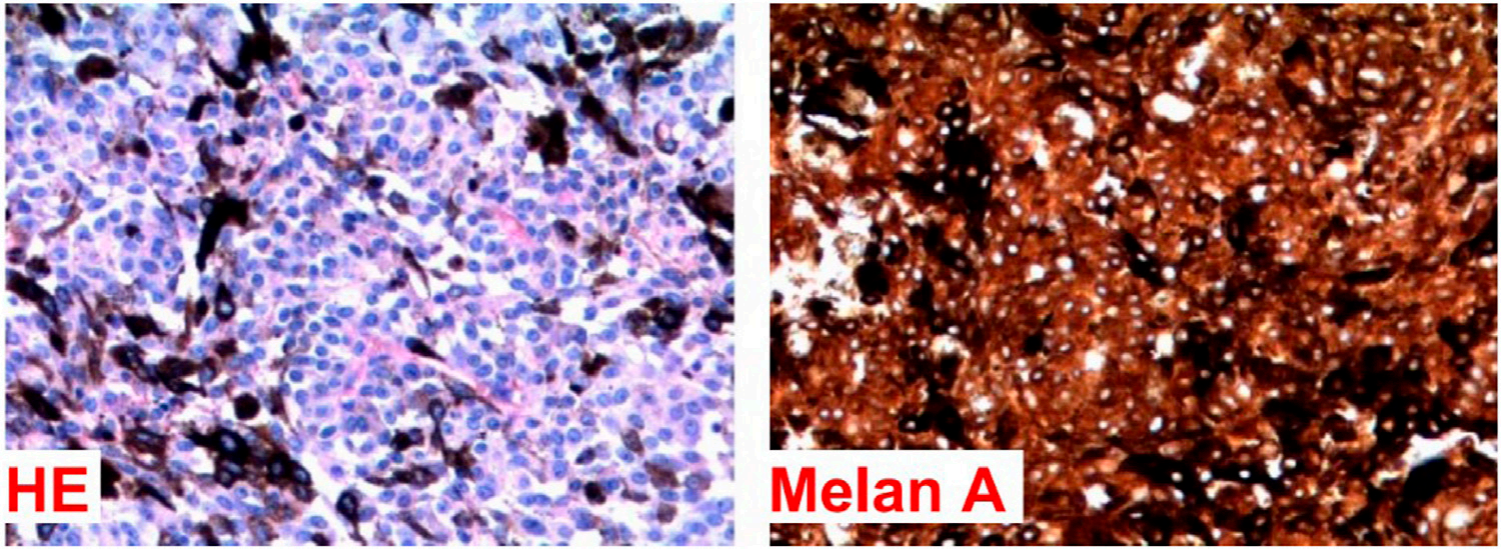
Histological examination of the lesion. Stained with HE and melan A.

The histology revealed a neoplasm characterized by spindle-shaped and epithelioid cells, featuring nuclei with vesicular chromatin, occasionally evident nucleoli, and amphophilic cytoplasm (see Figure 3). These tumor cells were arranged in fascicles or hypercellular aggregates. Additionally, melanophages containing melanin pigment in the cytoplasm were present. A low mitotic activity was observed, with fewer than 1 mitosis per 10 high magnification fields on average.

Histological characteristics of aggressiveness, such as nuclear atypia, necrosis, and invasion of healthy parenchyma, were not significantly found in the sample. These characteristics, together with the other features described, favor the diagnosis of an intermediate-grade melanocytoma rather than a primary melanoma of the leptomeninges.

A complementary immunohistochemical study (see Table 2) exhibited the expression of melan-A and SOX-10, while collagen IV deposition was absent. The Ki-67 index was also 1%. These findings suggest a primary melanocytic tumor of the central nervous system, with the diagnostic hypothesis being a primary leptomeningeal melanocytoma.

**TABLE 2:** Lesion immunohistochemical profile.

Antibody	Result
Sox-10	+
Ki-67	1%
Melan A	+
BRCA-1	Preserved expression

### Follow up

The patient was referred to the oncology department, where she underwent a follow-up MRI 3 months after surgery, revealing no signs of recurrence or residual tumor. Subsequently, she received conformal irradiation of the surgical bed, involving a total of five sessions.

Throughout her follow-up, an additional MRI was conducted 1 year after the surgery, demonstrating once again the absence of recurrence or residual tumor. The patient continues to undergo rigorous outpatient follow-up.

## Discussion

In this paper, we present the case of a 32 year-old female patient with two left-sided supratentorial lesions, initially suggestive of a convexity meningioma. However, standard histology indicated a melanocytic tumor without further specification. Complementary immunohistochemistry supported the diagnosis of primary melanocytoma in the leptomeningeal region.

The first reported case of meningeal melanocytoma in the CNS dates back to 1972 when Limas et al. documented it [16]. These solitary lesions are estimated to occur in approximately 1 in 10 million individuals annually. They tend to affect women during adulthood, often causing compression without invasive penetration into the adjacent brain tissue. The prognosis generally appears favorable, although instances of local recurrence are not uncommon [10, 13, 14].

Meningeal melanocytoma cases commonly appear in the cervical and thoracic spinal cord and the posterior fossa [13]. Supratentorial lesions, however, are exceedingly rare. Beseoglu et al. highlighted in their 2006 publication that only 25 supratentorial cases had been documented in the literature at that time [10]. The largest series of cases reported includes 14 instances with a varied age range of 27–69 years, an equal gender distribution, eight cases located in the posterior fossa, two cases in Meckel’s cavum, two cases in the cerebellopontine angle, and just one case in a convex region [17]. Two instances of convex lesions mimicking convex meningiomas were described: one in 2012 by Lin et al. [11] and another in 2006 by Beseoglu et al. [10].

Diagnosing primary melanocytoma of the leptomeninges involves imaging, biopsy, and immunopathological tests. On MRI, melanocytomas manifest as solitary masses that appear hyperintense or isointense on T1-weighting and hypointense or isointense on T2-weighting, accompanied by uniform contrast enhancement. In our case, these lesions were presented as two entities—one more superficial and the other deeper—where the latter appeared as a blood clot during surgery.

The main differential diagnoses that should be considered when an extra-axial lesion is found, with low-grade spindle cells, are meningioma, melanocytic schwannoma, and psammomatous schwannoma. As a result, neuroimaging cannot differentiate meningeal melanocytoma from other neoplasms with similar characteristics, particularly meningiomas, schwannomas, and malignant melanomas. The definitive diagnosis can only be established through histopathological study, often requiring complementation with an immunohistochemical analysis [13].

Primary melanocytes of the leptomeninges are considered low-grade tumors, with a slow growth rate of less than 1/10 hpf, little atypical cytology, and a lower chance of metastasis compared to primary melanomas of the leptomeninges. In addition, the cells are well-differentiated, with a spindle-shaped morphology, an oval and eosinophilic nucleus.

Despite the benign characteristics, if the melanocytoma has a high rate of mitosis or is invading adjacent tissue, it is classified as an intermediate-grade primary melanocytic neoplasm of the leptomeninges, which was the case found in this study [5]. In the assessment with the electron microscope, it is possible to observe melanosomes, absence of desmosomes, interdigitations, and basal lamina, characteristics that will make it possible to differentiate the melanocytoma from other lesions, such as meningioma and schwannoma [12].

Immunohistochemically, primary CNS melanocytomas will show the HMB-45, S-100, and vimentin antigens but will not show the EMA, GFAP, and Leu7 antigens. The performance and interpretation of immunohistochemistry will be important in differentiating melanocytomas from other types of pigmented tumors of the nervous system [18].

In genetic aspects, cutaneous, leptomeningeal, uveal, and mucosal melanocytic tumors are heterogeneous diseases with some molecular characteristics that distinguish them, making it possible to differentiate them through genetic mapping, which is useful for treatment and assessing prognosis [5]. Genetic alterations that increase the expression of the BRAF gene, responsible for synthesizing the serine/threonine protein kinase, are associated with an increase in the MAPK pathway, leading to increased cell proliferation [9].

Mutations in the BRAF gene are quite rare in primary melanocytic lesions of the CNS and in uveal melanomas; on the other hand, they are present in almost 50% of melanomas of cutaneous origin, so the presence of a mutation in this gene in a melanocytic lesion of the nervous system favors the diagnosis of a metastatic tumor over a primary CNS lesion [12].

In addition to BRAF, mutations in the TERT promoter genes, which synthesize telomerase, and in the NRAS gene, which is responsible for the formation of GTPases, are frequently found in cutaneous melanomas but rarely in primary melanomas of the leptomeninges [1].

On the other hand, alterations in the GNAQ and GNA11 genes, associated with the synthesis of the alpha subunit of the G protein, are uncommon in cutaneous melanomas, being present in less than 3% of them, but they are common in primary melanocytic lesions of the leptomeninges and in uveal melanomas, in which they are present in 56% and 77% of cases, respectively. Therefore, melanocytic lesions with this type of modification are unlikely to be associated with cutaneous melanoma metastasis [5, 19].

From both a therapeutic and prognostic standpoint, distinguishing meningeal melanocytoma from primary or metastatic CNS melanoma is of paramount importance. This is particularly relevant because the CNS serves as a common site for melanoma metastasis, often with the primary source being challenging to detect [10]. It’s widely agreed upon that complete surgical resection represents the optimal therapeutic approach. Even when complete removal is not achievable due to factors like the location or extent of tumor invasion, maximal partial resection should be pursued, followed by adjuvant radiotherapy sessions [15].

The utilization of radiotherapy in these lesions is still a topic of debate in the literature because, despite the benign nature of the tumor, recurrence rates for this type of lesion in patients who have undergone complete and incomplete resections can reach up to 71% within 5 years and 50% within 1 year, respectively [12].

Some authors recommend adjuvant radiotherapy in cases of both complete and partial resection of the lesion [13, 15]. In general, the current recommendation entails performing complete tumor removal, maintaining vigilant follow-up, whether or not adjuvant radiotherapy is pursued. In cases of recurrence, whenever possible, re-operation should be considered. If not feasible, referral for Radiosurgery is advised.

## Conclusion

Primary meningeal melanocytomas are rare lesions, predominantly documented in the medulla and skull base. Their occurrence in the supratentorial region is seldom reported in the literature. We present a case involving two lesions in the temporal region, initially resembling convexity meningiomas. The conclusive diagnosis was achieved solely through histological and immunohistochemical analyses.

Despite their benign character and low proliferation rate, local recurrence even after total resection remains a significant concern. Hence, the decision regarding adjuvant radiotherapy remains debatable in the literature. In our case, we opted to refer the patient for irradiation of the surgical bed, leading to no recurrence signs identified during over a year of follow-up.

Imaging examinations lack the capability to definitively diagnose these lesions, often leading to confusion with more prevalent tumors like meningiomas, schwannomas, and melanomas. The primary differential diagnosis lies with malignant melanoma, an exceptionally aggressive condition demanding more intensive treatment approaches.

## Data Availability

The raw data supporting the conclusion of this article will be made available by the authors, without undue reservation.
